# Infected Cardiac Myxoma: an Updated Review

**DOI:** 10.5935/1678-9741.20140112

**Published:** 2015

**Authors:** Shi-Min Yuan

**Affiliations:** 1 The First Hospital of Putian, Teaching Hospital, Fujian Medical University, Putian, China.

**Keywords:** Embolism, Infection, Myxoma

## Abstract

**OBJECTIVE:**

This study aims to present an updated clinical picture of the infected cardiac myxoma. Revankar & Clark made a systematic review of infected cardiac myxoma based on the literature before 1998. Since then, there has not been any updated information describing its recent changing trends.

**METHODS:**

A comprehensive literature search of infected cardiac myxoma was conducted on MEDLINE, Highwire Press and Google between 1998 and 2014.

**RESULTS:**

In comparison with Revankar & Clark's series, the present series disclosed a significantly decreased overall mortality. It is believed that refinement of the prompt diagnosis and timely management (use of sensitive antibiotics and surgical resection of the infected myxoma) have resulted in better outcomes of such patients.

**CONCLUSION:**

The present series of infected cardiac myxoma illustrated some aggravated clinical manifestations (relative more occasions of high-grade fever, multiple embolic events and the presence of refractory microorganisms), which should draw enough attention to careful diagnosis and treatment. In general, the prognosis of infected cardiac myxoma is relatively benign and the long-term survival is always promising.

## INTRODUCTION

Cardiac myxomas are rare. The classical clinical manifestations of cardiac myxomas can be a triad with constitutional, obstructive and embolic symptoms^[1]^. The infected cardiac myxoma is a very rare condition with only sporadic cases reported in the literature. In 1998, Revankar & Clark^[2]^ presented a complete literature collection of infected cardiac myxoma with a total of 40 cases, with detailed descriptions of the clinical features of this rare condition. They found no clear distinction between infected and uninfected cardiac myxomas, however, the infected myxomas are associated with more febrile symptoms and a higher risk of embolic events. In order to highlight the recent trends of infected cardiac myxoma, an updated review is made based on a renewed literature collection.

## METHODS

A comprehensive literature search was conducted on MEDLINE, Highwire Press and Google between 1998 and 2014. The search terms included "myxoma", "heart", "heart valve", "endocarditis", "infected", "bacteremia", "blood culture" and "sepsis". Data were extracted from the text, figures and tables, with details of the study population, demographics, onset symptom, duration of disease, risk factor, complication, microorganism, antibiotic use and surgical treatment, timing of surgical operation, follow-up duration and main outcomes including survival, postoperative complication, requirement of further surgical procedure and mortality.

Reference values of white blood cell count, hemoglobin, erythrocyte sedimentation rate and C-reaction protein were 4.0-10.0×10^9^/L, >12 g/dL (>11 g/dL for females), <20 mm/h and 0-1 mg/dL, respectively.

Quantitative data were presented as mean±standard deviation with range and median values, and intergroup differences were compared using the unpaired *t* test. Frequencies were compared using Fisher's exact test. Results with *P*<0.05 were considered statistically significant.

### Patients' information

A total of 39 reports^[3-41]^ in terms of infected cardiac myxoma were collected and these involved 39 patients. Their information was listed in Table 1. According to the diagnostic criteria of infected cardiac myxoma, 34 (87.2%) were definite^[3-36]^, 4 (10.3%) were probable^[37-40]^ and 1 (2.6%) was possible^[41]^, with a compatible distribution of the three-level infected cardiac myxomas reported by Revankar & Clark^[2]^. The locations of the infected cardiac myxomas were left atrium 27 (69.2%), right atrium 5 (12.8%), mitral valve 5 (12.8%), right ventricle 1 (2.6%) and unstated 1 (2.6%), also similar to the percentages of the locations reported by Revankar & Clark^[2]^. The only difference was a lack of biatrial myxomas in the present series but with multiple left atrial myxomas instead. There were 23 (62.2%) males and 14 (37.8%) females (*x*^2^=4.4, *P*=0.062) with a male-to-female ratio of 1.64, while gender of 2 patients was not given. Their ages were 49.2±15.7 (range, 12-74; median,52) years *(n*=37). No age difference was found between male and female patients (50.7±11.4 years *vs.* 46.9±21.3 years, *P*=0.4786).

**Table 1:** Summary of 39 patients with infected cardiac myxoma

Serial N^o^	Year	Author	Sex	Age	Microorganism	Diagnostic means	Location of myxoma	Operation	Outcome
Definite									
1	2011	Adamira	F	58	*Staphylococcus* & *Enterococcus faecalis*	TTE,TEE	LAx2	Yes	Survived
2	2006	Awab	M	58	*Histoplasma capsulatum*	TTE	LA	Yes	Survived
3	2011	Belgi Yildirim	M	47	*Streptococcus viridans*	TTE	LA	Yes	Survived
4	2010	Bhanot	M	54	*Staphylococcus lugdunensis*	CT, TEE	LA	Yes	Survived
5	2001	Brazill	M	52	*Streptococci viridans*	TTE, TEE	LA	Yes	Survived
6	2007	Chan	M	44	*Streptococcus viridans*	TTE	RV	Yes	Survived
7	2011	Chang	M	49	*Streptococcus bovis*	TTE, TEE	LA	Yes	Survived
8	2001	Dekkers	M	40	*Streptococcus mutans*	TTE, TEE	LA	Yes	Survived
9	2008	Falasca	F	63	*Staphylococcus spp*	TTE	LA	Yes	Survived
10	2013	Furukawa	M	62	*Streptococcus agalactiae*	TTE, TEE	LA	Yes	Survived
11	2005	García-Quintana	F	58	*Streptococcus oralis*	TTE	LA	Yes	Survived
12	2004	Gregory	M	43	MSSA	TTE (-), TEE (+)	LA	Yes	Survived
13	2007	Guler	F	12	MSSA	TTE	MV (AML)	Yes	Survived
14	2008	Janion	M	67	MRSA	TTE	LA	Yes	Survived
15	2005	Juang	M	42	G-positive cocci	TEE	LA	Yes	Survived
16	2004	Karachalios	M	55	*Streptococcus viridans*	TTE	LA	Yes	Survived
17	2007	Leone	F	74	*Enterococcus faecalis*	TTE	LA	Yes	Survived
18	2013	Nagata	M	66	*Streptococcus mitis*	TEE	LA	Yes	Survived
19	2001	Oyama	M	57	MRSA	TTE	LA	Yes	Survived
20	2004	Parks	M	36	*Staphylococcus aureus *(oxacillin-resistant) (ORSA)	TTE	RA	Yes	Survived
21	2002	Prince	F	12	*Streptococcus viridans*	TTE	LA	Yes	Survived
22	2001	Puvaneswary	M	50	*Streptococcus viridans & Streptococcus salivarius*	TTE (-), TEE (+)	RA	Yes	Survived
23	2006	Quigley	M	55	*Streptococcus mutans*	TEE	LA	Yes	Survived
24	2005	Riad	M	36	MRSA	TTE	RA	Yes	Survived
25	2010	Sasaki	M	69	*Klebsiella pneumoniae*	TEE	LA	Yes	Survived
26	2010	Surovcík	F	59	*Enterococcus faecalis*	TTE, TEE, CT	LA	Yes	Survived
27	2002	Tanaka	F	70	*Enterococcus faecalis*	TEE	LA	Yes	Survived
28	1999	Toda	M	20	Bacterial colonies	TTE	MV (PML, posterior commissure)	Yes	Survived
29	2009	Trimeche	F	46	MSSA	TTE, TEE	LA	NG	Died
30	2002	Uchino	F	47	*Streptococcus bovis*	TTE	LA	NG	Survived
31	2006	Veitch	F	35	*Staphylococcus aureus*	TTE, TEE	MV (AML)	Yes	Survived
32	2011	Yoshioka	M	52	MSSA	TTE	LA	Yes	Survived
33	2006	Zechini	M	55	Catalase-negative microorganisms (G-positive cocci by staining)	TTE, TEE	LA	NG	Survived
34	2013	Zwinkels	?		*Streptococcus* (Group C)	TTE, cardiac CT angiogram	RA	Yes	Survived
Probable									
35	2007	Bernstein	F	60	*Streptococcus mitis*	TEE	MV (PML)	Yes	Survived
36	2005	Liu	F	12	*Neisseria lactamica*	TTE	MV (AML)	Yes	Survived
37	1998	Marshall	F	50	*Actinobacillus* *actinomycetemcomitans*	TTE	LA	Yes	Survived
38	2001	Pinede	?		*Streptococcus viridans*	?	?	Yes	Survived
Possible									
39	2005	Despott	M	57	*Klebsiella pneumoniae*	TTE	RA	No	Died of right lung abscess

AML=anterior mitral leaflet; CT=computed tomography; F=female; LA=left atrium; M=male; MRSA=methicillin-resistant Staphylococcus aureus; MSSA=methicillin-sensitive Staphylococcus aureus; MV=mitral valve; NG=not given; PML= posterior mitral leaflet; RA=right atrium; TEE=transesophageal echocardiography; TTE= transthoracic echocardiography

### Risk factors

Recent dental procedures, recent infections, previous invasive procedures and immunocompromised conditions were the risk factors that led to a cardiac myxoma infected. No predominance was noted between the above risk factors. No significant difference was found in each risk factor between the present series and the series of Revankar & Clark^[2]^ (Table 2).

**Table 2:** Risk factors.

Risk factor	Present	Revankar & Clark's	*x*^2^	*P* value
Recent dental problem	3 (7.7)	9 (22)	3.4	0.115
Dental surgery	1			
Reconstructive dental procedures; coronary catheterization and angioplasty 2 years earlier	1			
Dental decay	1			
Recent infection	4 (10.3)	4 (10)	0.0	1.000
Achilles tendon infection	2			
Urinary tract infection	1			
Cellulitis, web-space abscess of hand	1			
Invasive procedure	4 (10.3)	2 (5)	0.8	0.432
Umbilical hernia repair	1			
Amputation above knee for intractable osteomyelitis	1			
Multiple surgery, closed trauma of the left knee	1			
Acupuncture for weight reduction	1			
Immunocompromised condition	5 (12.8)	3 (7.5)	0.6	0.481
Intravenous drug use, hepatitis C infection	2			
Diabetes mellitus	1			
Breast cancer (surgery, radiotherapy, chemotherapy), pharyngitis	1			
Traveled to Mexico 3 months before, patent fossa ovalis	1			
Total	16 (41.0)	18 (45)	0.1	0.821

### Clinical features

The onset symptoms and signs of the 37 patients (2 patients' symptoms were not reported) with infected cardiac myxoma were listed in Table 3. Fever was the most common symptom accounting for 97.3% of all cases. The fever nature was persistent in 2 (5.6%), recurrent in 1 (2.8%), intermittent in 1 (2.8%) and with a fever of unknown origin in 3 (8.3%) patients. In general, constitutional symptoms were more frequent than obstructive or neurological symptoms. The duration of symptoms before admission was 1.6±1.7 (range, 0.1-6; median, 1) months (*n*=21).

**Table 3:** Onset symptoms and signs of the patients with infected cardiac myxoma

Feature	Present	Revankar & Clark's	*x*^2^	*P* value
Symptom				
Fever	36 (97.3)	37 (92)	0.9	0.616
Embolic events	10 (27.0)	5 (12)	2.6	0.151
Weight loss	9 (24.3)	13 (32)	0.6	0.460
Dyspnea	8 (21.6)	3 (8)	3.1	0.106
Fatigue	8 (21.6)	11 (28)	0.4	0.605
Neurologic symptoms	7 (18.9)	8 (20)	0.0	1.000
Malaise	6 (16.2)	11 (28)	1.4	0.279
Rigor/shivers/chills	6 (16.2)	11 (28)	1.4	0.279
Night sweat	5 (13.5)	11 (28)	2.3	0.165
Weakness	3 (8.1)	5 (12)	0.4	0.713
Abdominal pain	3 (8.1)	3 (8)	0.0	1.000
Anorexia	3 (8.1)		3.4	0.106
Edemas	3 (8.1)		3.4	0.106
Chest pain/distress	2 (5.4)		2.2	0.228
Cough	2 (5.4)	4 (10)	0.6	0.676
Nausea/vomiting	2 (5.4)	1 (2)	0.4	0.605
Headache	1 (2.7)	4 (10)	1.7	0.360
Arm pain	1 (2.7)		1.1	0.481
Arthralgia	1 (2.7)	6 (15)	3.5	0.110
Diarrhea	1 (2.7)	3 (8)	0.9	0.616
Hemoptysis	1 (2.7)		1.1	0.481
Jaundice	1 (2.7)		1.1	0.481
Lethargy	1 (2.7)		1.1	0.481
Back pain	1 (2.7)		1.1	0.481
Myalgias	1 (2.7)		1.1	0.481
Palpitation	1 (2.7)		1.1	0.481
Septic shock	1 (2.7)		1.1	0.481
Sign				
Temperature (°C)				
<37.8	5/28 (17.9)	3/32 (9)	0.9	0.454
37.8-38.9	9/28 (32.1)	19/32 (59)	4.5	0.042
>38.9	14/28 (50)	10/32 (31)	2.2	0.189
Heart murmur	18 (48.6)	26 (65)	2.1	0.172
Loud S,	3 (8.1)	14 (35)	8.1	0.006
Extra heart sound		9 (22)	9.4	0.002
"Tumor plop"		2 (5)	1.9	0.494
Splenomegaly		3 (8)	2.9	0.241
Skin lesions	3 (8.1)	6 (15)	0.9	0.484
Neurological deficits	2 (5.4)	3 (8)	0.1	1.000

Laboratory findings showed that leukocyte count was elevated in 20 patients (76.9%) and normal in 6 (23.1%) patients (*x*^2^=15.1, *P*=0.000) with a quantitative result of 15.7±7.3 (range, 4.9-33.6; median, 15.2) ×10^9^/L (*n*=26). Hemoglobin was reduced indicating a mild anemia in 13 (92.9%) patients and normal in 1 (7.1%) patient (*x*^2^=20.6, *P*=0.000) with a quantitative result of 10.4±1.3 (range, 9-13.7; median, 10.8) g/dL (*n*=14). Erythrocyte sedimentation rate and C-reactive protein were elevated in all studied cases: erythrocyte sedimentation rate was 83.3±28.4 (range, 37-124; median, 83) mm/h (*n*=15) and C-reaction protein was 15.8±10.5 (range, 3-39.9; median, 13.2) mg/dL (*n*=17). Microscopic hematuria was present in 2 (5.4%) patients.

### Microorganisms

All 39 patients had microbiological evidence confirmed by one or more investigations including blood culture, culture of resected myxoma, histopathological inspection for bacteria or confirmation by polymerase chain reaction. Blood culture for microbiological isolation was positive in 37, negative in 1 and unstated in 1. Cultures of resected myxoma tissues were done in 15 patients: 9 (60%) were positive and 6 (40%) were negative. Histopathological studies of the resected myxomas were performed in 30 patients. They were histopathologically inspected for bacteria in 14 patients: 13 (92.9%) were positive and 1 (7.1%) was negative. The 41 bacteria of 39 patients were summarized in Table 4.

**Table 4:** Pathogens of infective cardiac myxoma

Pathogens	Present	Revankar & Clark's	*x*^2^	*P* value
*Streptococci*	17 (41.5)	20 (50)	0.6	0.507
* Streptococcus viridans*	7			
* Streptococcus mutans*	2			
* Streptococcus bovis*	2			
* Streptococcus mitis*	2			
* Streptococcus* (Group C)	1			
* Streptococcus agalactiae*	1			
* Streptococcus oralis*	1			
* Streptococcus salivarius*	1			
*Staphylococci*	12 (29.3)	7 (18)	1.6	0.295
MSSA	4			
MRSA	3			
* Staphylococcus aureus*	2 (1 was oxacillin-resistant (ORSA))			
* Staphylococcus lugdunensis*	1			
* Staphylococcus spp*	1			
* Staphylococcus* (species not given)	1			
*Enterococcus faecalis*	4 (9.8)	2 (5)	0.7	0.675
Gram-negative bacilli	2 (4.9)	3 (8)	0.2	0.675
* Klebsiella pneumoniae*	2			
Gram-negative cocci	1 (2.4)		1.0	1.000
* Neisseria lactamica*	1			
Fungus	1 (2.4)	3 (8)	1.1	0.359
* Histoplasma capsulatum*	1			
Actinomyce	1 (2.4)		1.0	1.000
*Actinobacillus actinomycetemcomitans*	1			
Unknown	3 (7.3)	3 (8)	0.0	1.000
Bacterial colonies	1			
Gram-positive cocci	2			

Histopathology showed inflammatory cell infiltrate in 11 (36.7%), necrosis in 4 (13.3%) and micro-/focal abscesses in 2 (6.7%) patients, respectively.

### Complications

There were 12 patients in whom complications developed. Of these, embolic events in 10 (8 were multiple sites or multiple organs), sepsis in 4 (one was septic shock), disseminated intravascular coagulation in 3 and lung abscess in 1. The embolic events were shown in Table 5. In comparison, 18 patients of Revankar & Clark's^[2]^ series developed embolic events and only one of them were multisites (80% (8/10) *vs.* 5.6% (1/18), *x*^2^=16.3, *P*=0.000).

**Table 5:** Embolic events.

Location	*n* (%)
Cerebral	1 (10)
Cerebral, peripheral (extremities)	1 (10)
Cerebral, splenic	1 (10)
Cerebral, splenic, renal	1 (10)
Coronary (left anterior descending coronary artery)	1 (10)
Multiple (location not indicated)	1 (10)
Multiple peripheral (left common + external iliac arteries + right deep femoral artery)	1 (10)
Pulmonary	1 (10)
Splenic	1 (10)
Splenic, renal	1 (10)

### Treatment

Thirty-eight of 39 patients received a surgical treatment of infected cardiac myxoma and the reason that only patient did not undergo any surgical procedure was due to rapid deterioration leading to death. Of the 38 surgical operations, 30 were isolated cardiac myxoma resections, 5 were cardiac myxoma resection with concurrent cardiac surgical procedures (mitral valve replacement in 2, and mitral valve replacement + coronary artery bypass grafting, mitral valve replacement + embolectomy of the left anterior descending coronary artery + coronary artery bypass grafting, and pulmonary valve replacement in 1 patient each) and 3 with staged peripheral operations (3^rd^ toe of right foot amputation & right femoropoplitereal bypass; right common iliac artery thrombectomy and embolectomy of the left common and external iliac and right deep femoral arteries in 1 patient each).

The timing for surgical operation was reported in 26 patients, 4 of them were operated on an urgent basis and 22 had a delay of 18±13.5 (range, 3-42; median, 14) days (*n*=21) after admission for the purpose of sufficient preoperative antibiotic treatment and stabilization of patients' conditions. Preoperative antibiotic treatment was described in 26 patients. The frequently used antibiotics included vancomycin (7 patients), penicillin (4 patients) and ampicillin (3 patients). Sometimes, they were used along with gentamicin (80 mg every 8 h). Other antibiotics were imipenem, meropenem, peperacillin, trimethoprim/sulfamethoxazole, oxicillin, ampicillin, nafcillin, teicoplanin, azitromicine, ceftriaxone, clindamycin and amoxicillin-clavulanic acid.

The duration of preoperative antibiotic use was 22.3±13.4 (range, 7-42; median, 18) days (*n*=12). Postoperative antibiotic regimens included vancomycin (500 mg every 6 h), ceftazidime (2 g every 8 h) and netilmicin (150 mg every 12 h) followed by cefepime (1 g every 12 h) plus teicoplanin (400 mg daily), nafcillin (2 g intravenously every 6 h) and gentamicin and antifungal drugs with a therapeutic course of 31.2±5.1 (range, 27-42; median, 28) days (*n*=9).

### Outcomes

**Fig. 1:**
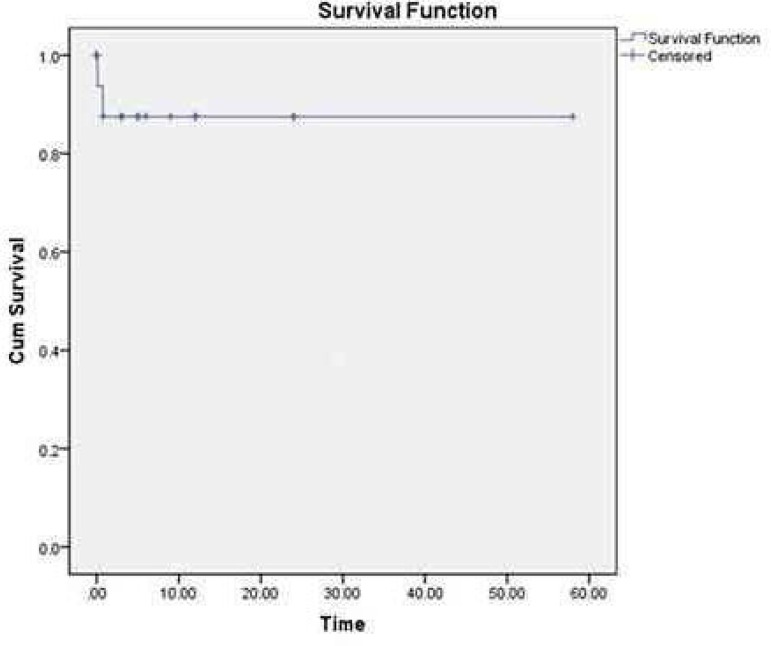
The overall survival of the present series was 92.6%.

**Fig. 2:**
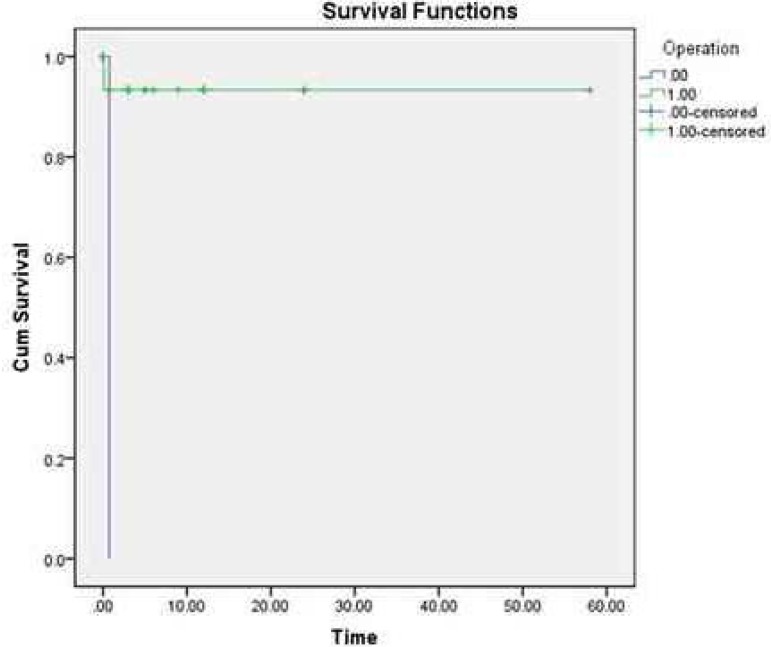
The survival rates of the surgical and non-surgical patients were 96.2% and 0%, respectively.

During a follow-up period of 11.1±14.5 (0.1-58; median, 8.5) months (*n*=16), 37 (92.6%) patients survived and 2 (7.4%) died. Of the 37 survived patients, 35 (94.6%) were event-free and 2 (5.4%) were complicated with ruptured saccular abdominal aortic aneurysm with renal infarct and septic emboli requiring aortoiliac bypass in one patient, and increased cerebral hematoma requiring craniotomy in another. One patient died of rupture of a right lung abscess 3 weeks after admission and the other died of disseminated intravascular coagulation on postoperative day 10. The overall mortality was 5.1% and the operative mortality was 2.6%, with a significantly reduced overall mortality in comparison with that of the patient series of Revankar & Clark (overall mortality: 5.1% *vs*. 21%, *x*^2^=4.0, *P*=0.047; operative mortality: 3% *vs.* 2.6%, *x*^2^=1.4, *P*=0.239). The overall survival rate was 92.6% (Figure 1). The survival rates of the surgical and non-surgical patients were 96.2% and 0%, respectively (Figure 2).

## DISCUSSION

In 1998, Revankar & Clark^[2]^ defined infected cardiac myxoma in three levels based on clinical and pathological findings of the myxoma:

Definite infected cardiac myxoma

1. Documented myxoma by pathology and
2a. Microorganisms seen on pathology or
2b. Positive blood cultures and inflammation on pathology.

Probable infected cardiac myxoma

1. Documented myxoma by pathology and
2. Positive blood cultures or inflammation on pathology.

Possible infected cardiac myxoma

1. Characteristic appearance by transthoracic or transesophageal echocardiography and
2. Positive blood cultures.

Using these criteria, the three-level infected cardiac myxoma accounted for 85%, 12.5% and 2.5% respectively as reported by Revankar & Clark^[2]^, and 87.2%, 10.3% and 2.6% in the present series.

The diagnosis of infected cardiac myxoma can be a challenge. The time interval from symptom onset to establishment of the diagnosis varied from 0 to 126 (median, 4) months^[40]^. Pathogens can be evidenced by blood culture, culture or staining of resected myxoma, and occasionally confirmation by polymerase chain reaction is helpful. The transthoracic or transesophageal echocardiography is a reliable means for the detection of a cardiac myxoma. Only in patients with rapid deterioration leading to sudden death was the diagnosis of a cardiac myxoma established by autopsy.

Comparisons between the two series revealed that the present series were characterized by few occasions of moderate-grade fever (while more occasions of high-grade fever in spite of lack of a statistical significance) and abnormal heart sound, but more uncommon microorganisms and more severe complications -- multiple embolic events. Other clinical features of the two patient series did not differ from each other. The somehow aggravated situations may contribute to the presence of refractory microorganisms, such as Gram-negative bacteria, fungus and actinomyce.

In comparison with Revankar & Clark's^[2]^ series, the present series disclosed a significantly decreased overall mortality. It is believed that refinement of the prompt diagnosis and timely management (use of sensitive antibiotics and surgical resection of the infected myxoma) have resulted in better outcomes of such patients.

## CONCLUSION

In conclusion, the present series of infected cardiac myxoma illustrated somehow aggravated clinical manifestations (relative more occasions of high-grade fever, multiple embolic events and the presence of refractory microorganisms), which should draw sufficient attention to be careful in the diagnosis and treatment. In general, the prognosis of infected cardiac myxoma is relative benign and now the long term survival is always promising.

**Table:** 

**Authors' roles & responsibilities**	
SMY	Study conception and design; analysis and/or interpretation of data; manuscript writing, final approval of the manuscript.
